# MiRNA-190a-5p promotes primordial follicle hyperactivation by targeting PHLPP1 in premature ovarian failure

**DOI:** 10.3389/fgene.2022.1034832

**Published:** 2022-11-03

**Authors:** Yuchi Zhang, Dongwei Han, Xiaoyan Yu, Xinyu Shao, Chuju Zong, Manyu Zhang, Junzhi Wang, Jingwen Liang, Pengling Ge

**Affiliations:** ^1^ Department of Pharmacology, School of Basic Medical Sciences, Heilongjiang University of Chinese Medicine, Harbin, China; ^2^ Department of Neurology, Faculty of Medicine, Shimane University, Izumo, Japan; ^3^ The First Affiliated Hospital of Qiqihar Medical University, Qiqihar, China; ^4^ Heilongjiang Institute for Drug Control, Harbin, China; ^5^ Department of Dermatology, First Affiliated Hospital, Heilongjiang University of Chinese Medicine, Harbin, China

**Keywords:** premature ovarian failure (POF), 4-vinylcyclohexene diepoxide (VCD), miRNA-190a-5p, PHLPP1, AKT, LHR, FOXO3a

## Abstract

We previously screened 6 differentially expressed miRNAs in ovarian tissues of 4-vinylcyclohexene diepoxide (VCD)-treated premature ovarian failure (POF) model in SD rats, including miRNA-190a-5p, miRNA-98-5p, miRNA-29a-3p, miRNA-144-5p, miRNA-27b-3p, miRNA-151-5p. In this study, to investigate the mechanisms causing the onset of POF, we first identified miRNAs with earlier differential expression at consecutive time points in the VCD-treated rat POF model and explored the mechanisms by which the target miRNAs promote POF. The SD rats were injected with VCD for 15 days to induce POF. Additionally, we collected rat blood and ovaries at the same time every day for 15 consecutive days, and luteinizing hormone (LH), follicle-stimulating hormone (FSH), Anti-Mullerian hormone (AMH), and estradiol (E_2_) serum levels were detected by ELISA. Six miRNAs expression were measured in rat ovaries by qRT-PCR. Dual-luciferase reporter gene assays were employed to predict and verify the target gene (PHLPP1) of target miRNAs (miRNA-190a-5p). Western blot was examined to detect the expression levels of PHLPP1, AKT, p-AKT, FOXO3a, p-FOXO3a, and LHR proteins on the target gene PHLPP1 and its participation in the primordial follicular hyperactivation-related pathways (AKT-FOXO3a and AKT-LH/LHR). During the VCD modeling POF rat ovaries, miRNA-190a-5p was the first to show significant differential expression, i.e., 6th of VCD treating, and PHLPP1 was verified to be a direct downstream target of it. Starting from the 6th of VCD treatment, the more significant the up-regulation trend of miRNA-190a-5p expression, the more obvious the down-regulation trend of PHLPP1 and LHR mRNA and protein expression, accompanied by the more severe phosphorylation of AKT and FOXO3a proteins, thus continuously over-activating the rat primordial follicle to promote the development of POF. In conclusion, miRNA-190a-5p may become a potential biomarker for early screening of POF, and it can continuously activate primordial follicles in rats by targeting the expression of PHLPP1 and key proteins in the AKT-FOXO3a and AKT-LH/LHR pathways.

## 1 Introduction

With the change in people’s lifestyles and fertility concepts, women’s childbearing age is moving backward, and the incidence of infertility is increasing year by year (Harumi, 2009). According to the World Health Organization (WTO) reported, infertility will become the third largest disease after cancer and cardiovascular disease (Vayena et al., 2002; Dong et al., 2021). Meanwhile, the latest survey showed that the incidence rate of infertility in the world is 15% to 20%, and the number of patients ranges from 48 million to 180 million (Thoma et al., 2021). Among them, premature ovarian failure (POF) is one of the important pathogenic factors that cause infertility in women of childbearing age, and it is a gynecological disease with unclear etiology (Shelling, 2010). Most patients are found to be in a state of POF due to menstrual disorders, amenorrhea, and infertility (Shelling, 2010). However, at present, the onset time of POF cannot be determined, and it has the characteristics of being irreversible or difficult to reverse (Santoro, 2003). Thus, early detection in the initial process of POF is an urgent medical research problem.

With the deepening of medical research, more and more evidence has confirmed that the over-activation of primordial follicles is an important pathological mechanism for POF, and it is also caused by the premature depletion of ovarian reserves (Adhikari et al., 2013; Zhou et al., 2017; Chakravarthi et al., 2020; Maidarti et al., 2020). Therefore, it is particularly important to find out the regulatory factors of the over-activation of primordial follicles to explore the mechanism of POF. Studies have shown that a common ovarian toxic chemical, 4-vinylcyclohexene diepoxide (VCD), accelerates ovarian failure by specifically activating primordial follicles (Hu et al., 2006; Fernandez et al., 2008; Kappeler and Hoyer, 2012; Lee et al., 2017). In our previous research, we replicated the POF rat model by VCD (Li et al., 2014), and detected 6 differentially expressed miRNAs between POF rats and normal rats by rat genome-wide miRNA expression profile technology, including miRNA-190a-5p, miRNA-98-5p, miRNA-29a-3p, miRNA-144-5p, miRNA-27b-3p, miRNA-151-5p (Kuang et al., 2014). Then, we speculated that the 6 differentially expressed miRNAs might be involved in the occurrence of POF. However, it is unknown whether these differentially expressed miRNAs are the cause of excessive activation of primordial follicles in early premature ovarian failure or the result of hyperactivation of primordial follicles. Thus, this study will detect the expression of 6 miRNAs at a continuous time point from the first day of the application of VCD to replicate the POF rat model, identify the target miRNAs with early differential expression, and verify whether they can induce POF.

## 2 Materials and methods

### 2.1 Animals

Twelve weeks old female Sprague-Dawley (SD) rats, weighing 200 ± 20 g, were supplied by the Liaoning Changsheng Biotechnology Co., Ltd. (Benxi, China) and were housed in the Heilongjiang University of Chinese Medicine with SPF conditions. The rats were housed in a temperature (20 ± 1°C)-and humidity (50 ± 5%)-controlled animal facility and maintained on a 12-h light/dark cycle and were acclimatized for 1 week before the experiment and allowed free access to a rodent diet and tap water. All experiments were approved by the Animal Experimental Ethical Committee of Heilongjiang University of Chinese Medicine (Heilongjiang, China) and performed by the Guide for the Care and Use of Laboratory Animals.

### 2.2 Experimental design

**FIGURE 1:**
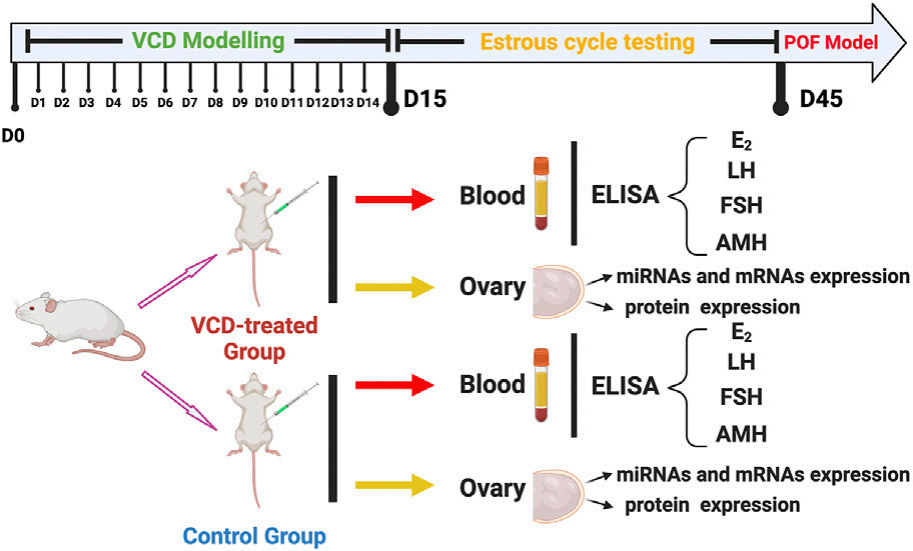
The experimental design of this study. The SD female rats divided into two groups: The VCD-treated group and Control group. The two group rats were sacrificed at the same time every day (D1-D15) for 15 consecutive days. After estrous cycle testing, the POF model rats also were collected. Each rat was detected with serum hormone levels (E_2_, LH, FSH, and AMH) and screened for early differential expression of target miRNAs from those significantly differentially expressed on POF model rats (miRNA-190a-5p, miRNA-98-5p, miRNA-29a-3p, miRNA-144-5p, miRNA-27b-3p, and miRNA-151-5p). Subsequently, ovarian tissues from rats on the 6th (D6), 10th (D10), 15th (D15), and 45th (D45) of VCD injection were selected to detect the expression levels of target genes of target miRNAs and mRNAs and proteins on the primordial follicle hyperactivation signaling pathway.

Two hundred female SD rats with an estrous cycle of 5 days were selected as experimental animals, we randomly divided them into two groups: The control groups (n = 100) and the VCD-treated groups (n = 100). Then the rats were intraperitoneally injected with sesame oil or sesame oil plus VCD (80 mg/kg/day) for 15 consecutive days according to our previous study (Kuang et al., 2014). At the same time, we sacrificed the rats in the control group and VCD-treated group every day (D1-D15, n = 6) for 15 consecutive days, and collected the blood and ovaries. Then we used the vaginal cytology method to observe the changes in the estrous cycle of rats. After 30 consecutive days of observation, the rats with estrous cycle disorder were taken as the POF model group rats. After 30 days of estrous cycle testing, we also collected the blood and ovaries of the D45 control group (n = 6) and the VCD-treated group (n = 6) rats. At the end of the experimental period, all rats were sacrificed by CO_2_ inhalation. All blood samples were centrifuged at 4,000 r/min and 4°C for 20 min to obtain blood serum samples for Enzyme-Linked Immunosorbent (ELISA) assays. After blood serum collection, all the ovaries were collected. From each rat, one ovary was rapidly frozen by liquid nitrogen and stored at 80°C for Quantitative real-time PCR (qRT-PCR), and the other ovary was stored at −80°C for Western blot analysis (Figure 1).

### 2.3 Enzyme-Linked Immunosorbent assays (ELISA)

The levels of rat serum estradiol (E_2_), anti-Mullerian hormone (AMH), follicle-stimulating hormone (FSH), and luteinizing hormone (LH) were determined by ELISA kits (Cloud-Clone Corp Inc. United States) following the manufacturer’s instructions. Briefly, LH, AMH, E_2_ or FSH standards at a final concentration of 8000, 2666.67, 888.89, 296.30, 98.77, and 0 pg/ml, 10000, 3333.3, 1111.1, 370.4, 123.5, and 0 pg/ml, 1000, 333.33, 111.11, 37.04, 12.35, and 0 pg/ml or 600, 200, 66.67, 22.22, 7.41, 2.4, and 0 ng/ml or diluted rat serum were added to anti-LH, AMH, E_2_ or FSH antibody-coated wells and incubated for 30 min. After washing five times, the horseradish peroxide (HRP)-conjugated detection antibodies were added, followed by the addition of the substrate solution. The optical density (OD) value was determined at a wavelength of 450 nm.

### 2.4 RNA extraction and quantitative real-time PCR (qRT-PCR)

According to the manufacturer’s instructions, total RNA was extracted from whole ovaries by TRIzol reagent (Invitrogen, United States) and miRNA or mRNA easy Minikit (Qiagen, Valencia, CA, United States). In addition, RNA concentrations were determined by NanoDrop ND-1000 spectrophotometer (Wilmington, DE, United States).

For qRT-PCR experiments, the RNA was reverse-transcribed into cDNA with a RevertAid First Strand cDNA Synthesis Kit (Thermo Fisher, United States) following the manufacturer’s instructions. Quantitative PCR reaction was run on a CFX Connect Real-Time PCR Detection Instrument (BioRad, United States). Expression of the selected miRNAs was quantified by qRT-PCR, after reverse transcription with miRNA-specific stem-loop primers. The primer sequences are shown in Tables 1, 2 to amplify fragments. The data were normalized to expression levels of the housekeeping genes U6 and GAPDH, respectively, and the 2^−ΔΔCT^ method was used to calculate relative expression levels.

**TABLE 1:** qRT-PCR primers of miRNAs.

Gene	Forward primer sequence [5′→3′]	Stem-loop primer sequence [5′→3′]	Accession ID	Primer melting temperature (T_m_, °C)
rno-miRNA-190a-5p	CGC​GCG​TGA​TAT​GTT​TGA​TAT​A	GTC​GTA​TCC​AGT​GCA​GGG​TCC​GAG​GTA​TTC​GCA​CTG​GAT​ACG​ACA​CCT​AA	NR_031906	77.5
rno-miRNA-144-5p	GCG​CGG​GAT​ATC​ATC​ATA​TAC​T	GTC​GTA​TCC​AGT​GCA​GGG​TCC​GAG​GTA​TTC​GCA​CTG​GAT​ACG​ACA​CTT​AC	NR_031890	78
rno-miRNA-27b-3p	CGGGTTCACAGTGGCTAA	CCT​GTT​GTC​TCC​AGC​CAC​AAA​AGA​GCA​CAA​TAT​TTC​AGG​AGA​CAA​CAG​GGC​AGA​AC	NR_031832	78
rno-miRNA-29a-3p	CGG​GTA​GCA​CCA​TCT​GAA​A	CCT​GTT​GTC​TCC​AGC​CAC​AAA​AGA​GCA​CAA​TAT​TTC​AGG​AGA​CAA​CAG​GTA​ACC​GA	NR_031836	77.2
rno-miRNA-98-5p	CGG​GGT​GAG​GTA​GTA​AGT​TG	CCT​GTT​GTC​TCC​AGC​CAC​AAA​AGA​GCA​CAA​TAT​TTC​AGG​AGA​CAA​CAG​GAA​CAA​TA	NR_031855	76
rno-miRNA-151-5p	CGGTCGAGGAGCTCACA	CCT​GTT​GTC​TCC​AGC​CAC​AAA​AGA​GCA​CAA​TAT​TTC​AGG​AGA​CAA​CAG​GAC​TAG​AC	NR_031798	78
U6	CCTGCTTCGGCAGCACA			80.3

Note: Universal reverse primer in rno-miRNA-27b-3p, rno-miRNA-29a-3p, rno-miRNA-98-5p and rno-miRNA-151-5p was: CAG​CCA​CAA​AAG​AGC​ACA​AT., The other universal reverse primer in rno-miRNA-190a-5p and rno-miRNA-144-5p was: AGT​GCA​GGG​TCC​GAG​GTA​TT., The universal reverse primer of U6 was AAC​GCT​TCA​CGA​ATT​TGC​GT.

**TABLE 2:** qRT-PCR primers of mRNAs.

Gene	Forward primer sequence [5′→3′]	Reverse primer sequence [5′→3′]	Accession ID	Primer melting temperature (T_m_, °C)	Product length
Phlpp1	AGA​CGC​CAG​GTC​ATT​CTG​TG	TTG​ACG​CAG​CCA​TCG​TAA​GT	NM_021657.1	60	216 bp
Akt1	CAA​GAT​GTG​TAT​GAG​AAG​AAG​CTG​A	GTT​CAC​TGT​CCA​CAC​ACT​CCA	NM_033230.2	60	154 bp
Foxo3a	GTC​ACG​ACA​AGT​TCC​CCA​GT	AGT​TTG​AGG​GTC​TGC​TTT​GCC	NM_001106395.1	60	261 bp
Lhr	TCG​CCC​TGT​CTT​CCT​ACT​CA	TGG​CGG​AAT​AAA​GCG​TCT​CG	NM_012978.1	60	213 bp
Gapdh	AGT​GCC​AGC​CTC​GTC​TCA​TA	GAT​GGT​GAT​GGG​TTT​CCC​GT	NM_017008.3	60	248 bp

### 2.5 Dual-luciferase reporter gene assay

Dual-Luciferase Reporter Gene Assay Target genes of rno-miRNA-190a-5p were predicted using target gene prediction software miRBase (http://www.mirbase.org) and TargetScan (http://www.targetscan.org/vert_71/). Plasmids containing wild-type PHLPP1 Luciferase reporter gene vector (PHLPP1) and mutant PHLPP1 Luciferase reporter gene vector (PHLPP1-mut) were constructed. They were co-transfected with rno-miR-190a-5p mimics or negative control (NC), respectively into 293T cells. Luciferase activity was detected by the Dual-Luciferase Reporter Assay System (Promega, Madison, WI) after 24 h.

### 2.6 Western blot analysis

Three frozen rat ovaries tissues in each group were homogenized in RIPA buffer with 1% Phenylmethylsulfonyl fluoride (PMSF). Then the homogenates were centrifuged at 12,000× g for 10 min, and the supernatants were collected for western blotting. 12 µg of total protein samples were separated on SDS-PAGE gels, transferred to PVDF membranes (0.45 μm, Millipore, IPVH00010), and blocked with 5% non-fat milk in TBS containing 0.1% Tween 20 (TBST). The membranes were incubated with the following primary antibodies: anti-PHLPP1, anti-AKT, anti-p-AKT, anti-FOXO3a, anti-p-FOXO3a, anti-LHR and anti-GAPDH (1:1000, Abcam, United Kingdom), diluted in TBST-5% milk at 4°C overnight. The appropriate horseradish peroxidase-conjugated secondary antibodies were diluted 1:1000 in TBST-5% milk and incubated for 1 h at room temperature, and the protein bands were visualized with enhanced chemiluminescence (ECL).

### 2.7 Statistical analysis

All results are represented as a mean ± standard error of the mean. Statistical analysis of these results was carried out by *t*-test or one-way ANOVA (with LSD or Dunnett’s T3 correction for comparison of multiple means). *p* values <0.05 were considered statistically significant. All statistical analyses were done using IBM SPSS 19.0 Software.

## 3 Results

### 3.1 Identification of miRNAs that are differentially expressed earlier in when VCD treatment process

**FIGURE 2:**
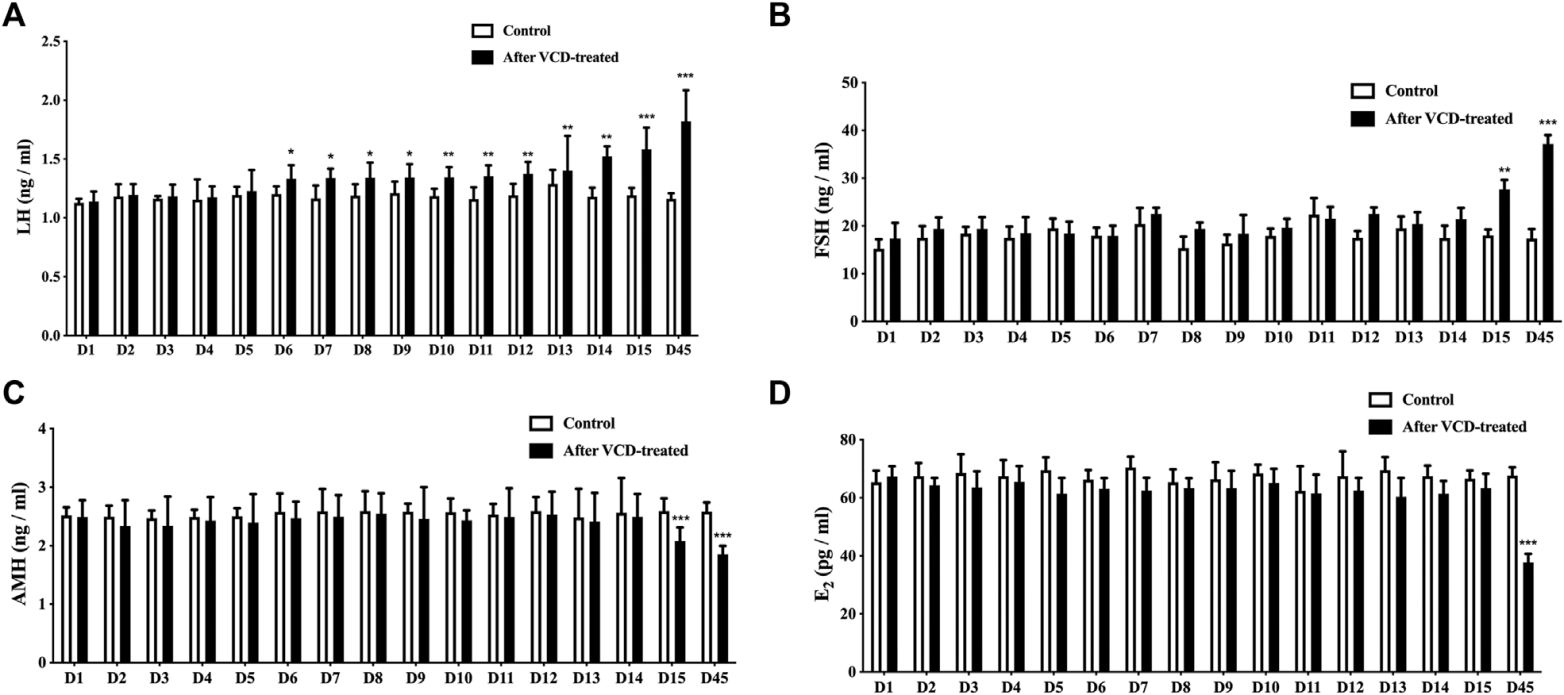
LH, FSH, AMH and E_2_ serum levels. **(A)**LH, **(B)**FSH, **(C)**AMH, and **(D)**E_2_ serum levels were detected by ELISA during the VCD-treated process (D1-D15, D45). Data are summarized and presented as mean ± SEM from four independent experiments. *p＜0.05, **p＜0.01 and ***p＜0.001 vs. control.

We first observed the changes of E_2_, FSH, LH, and AMH hormone levels in rat serum by ELISA to determine the development of POF in rats and whether it was successfully created or not. We found that with the increase of VCD-treated days (D1-D15), the LH levels increased significantly increasing the number of VCD-treatment days starting from the D6 VCD-treated group (*p* < 0.05, *p* < 0.01, *p* < 0.001, Figure 2A), the FSH level increased significantly in D15 VCD-treated group (*p* < 0.01, Figure 2B), the AMH level decreased significantly in D15 VCD-treated group (*p* < 0.001, Figure 2C) and there were no significant changes in E_2_ level (Figure 2D). On the 45th day (D45), which is the day to judge whether the POF animal model is successful or not, we found that the levels of LH and FSH level were significantly increased (*p* < 0.001, Figures 2A,B) and the levels of E_2_ and AMH level in the VCD-treated groups were significantly decreased (*p* < 0.001, Figures 2C,D).

**FIGURE 3:**
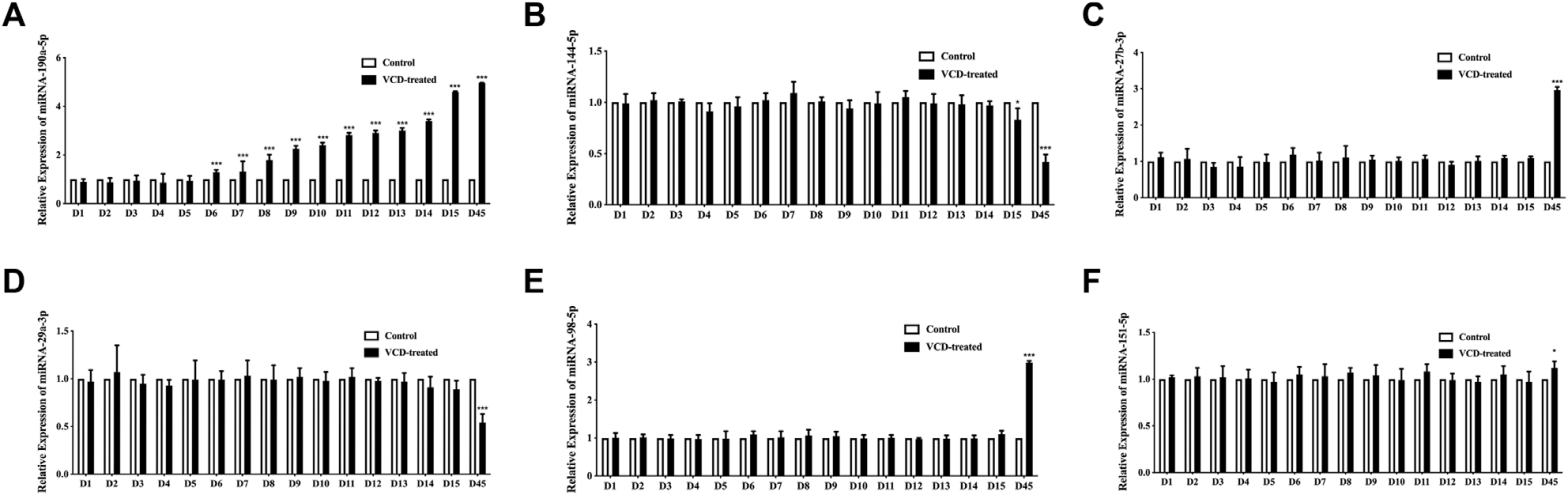
Screening of 6 miRNAs for early differential miRNAs in the VCD modeling process. **(A)** miRNA-190a-5p, **(B)** miRNA-144-5p, **(C)** miRNA-27b-3p, **(D)** miRNA-29a-3p, **(E)** miRNA-98-5p and **(F)** miRNA-151-5p expression levels during VCD-treated process (D1-D15, D45) in rat ovaries tissues were determined by qRT-PCR analysis. Data are summarized and presented as mean ± SEM from six independent experiments. *p＜0.05 and ***p＜0.001 vs. control.

Then we used qRT-PCR analysis in rat ovarian tissue to observe the 6 significantly differentially expressed miRNAs (miRNA-190a-5p, miRNA-27b-3p, miRNA-29a-3p, miRNA-98-5p, miRNA-151-5p, and miRNA-144-5p), which were being screened in the POF model rat ovary by our previous research, expression level changes during VCD-treated process (D1-D15). In this study, we found that miRNA-190a-5p was significantly differentially up-regulated at the earliest in the D6 VCD-treated group, with the increase of VCD-treated days, the up-regulation trend of differential expression became more and more significantly (*p* < 0.001, Figure 3A) compared with other miRNAs, the expression of miRNA-144-5p was up-regulated on the D15 VCD-treated group (*p* < 0.05, Figure 3B), and there was no significant difference in other miRNAs (Figures 3C–F). Meanwhile, this study also verified our previous findings. In the D45 VCD-treated group, miRNA-190a-5p, miRNA-27b-3p, miRNA-98-5p and miRNA-151-5p were significantly up-regulated (*p* < 0.001, Figures 3A,C,E,F), while miRNA-144-5p and miRNA-29a-3p were significantly down-regulated (*p* < 0.001, Figures 3B,D).

### 3.2 miRNA-190-5p directly targets PHLPP1 in the VCD-treating process

**FIGURE 4:**
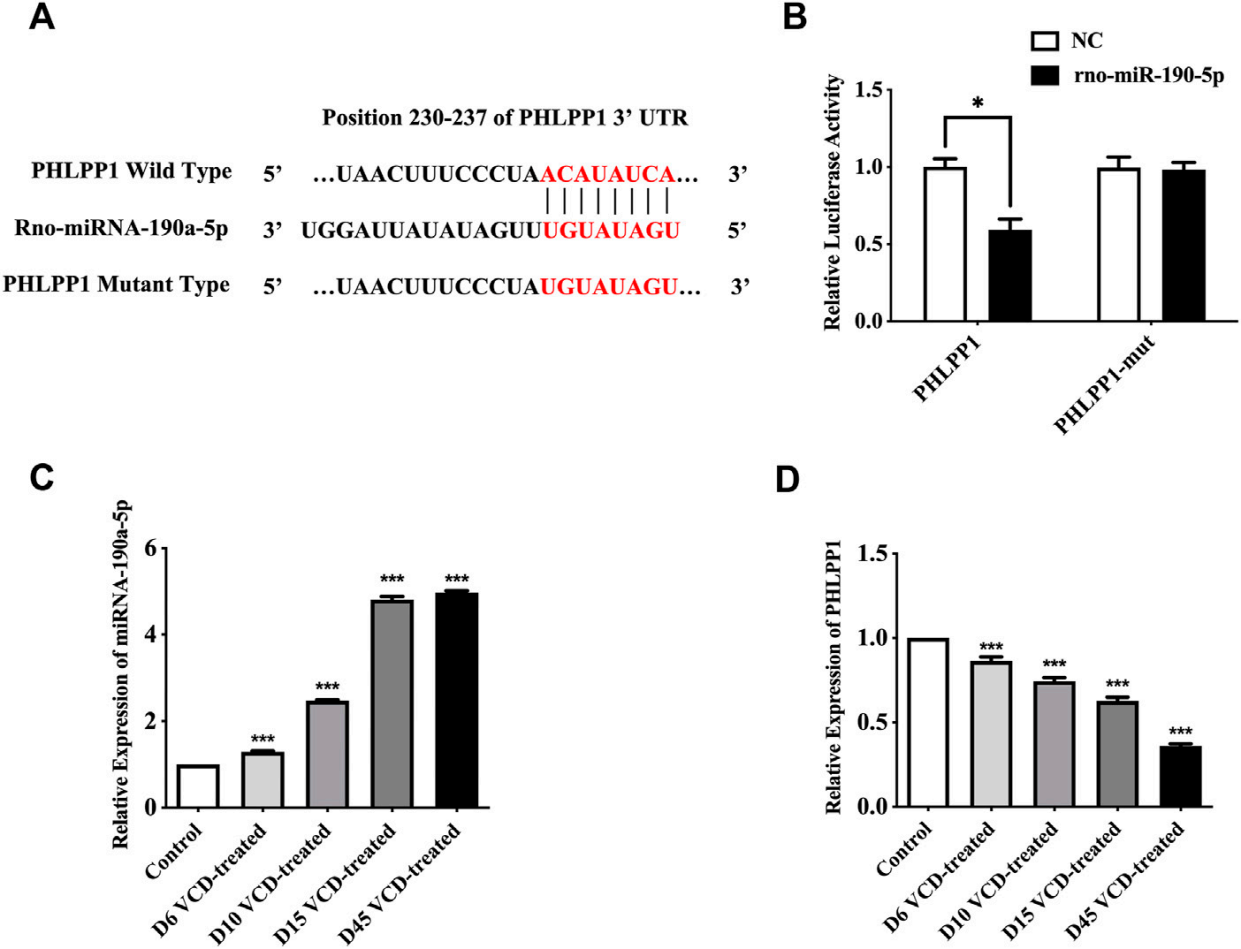
MiRNA-190-5p directly targets PHLPP1 during the VCD-treating process. **(A)** Diagram of putative miRNA-190a-5p binding sites of PHLPP1. Binding sequences for miRNA-190a-5p in the 3′-UTR of PHLPP1 and the mutations in the 3′-UTR of PHLPP1 are presented. **(B)** Luciferase activity of the wild-type PHLPP1 3′-UTR (PHLPP1) and mutant PHLPP1 3′-UTR (PHLPP1-mut) co-transfected with miRNA-190a-5p mimics (rno-miR-190a-5p) or negative control (NC) was measured. **(C)** and **(D)** qRT-PCR analysis of miRNA-190a-5p, PHLPP1 mRNA in rat ovaries tissues during the VCD treating process (D6, D10, D15, and D45). Data are summarized and presented as mean ± SEM from three independent experiments. ^*^ *p*＜0.05 vs. NC and ***p＜0.001 vs. control.

PHLPP1 was selected as a candidate miRNA-190a-5p target based on bioinformatics analysis (miRBase and TargetScan). As shown in Figure 4A, PHLPP1 was found to be a possible target gene of rno-miRNA-190a-5p. Then, the dual-luciferase reporter gene assay verified that miRNA-190a-5p impaired the luciferase activity of the wild-type PHLPP1 (PHLPP1) but not the mutant PHLPP1 3′-UTR (PHLPP1-mut) in cells (Figure 4B). The result suggested that PHLPP1 was a target of miRNA-190a-5p. We then selected rat ovarian tissues from the VCD-treated groups on the 6th (D6), 10th (D10), 15th (D15), and 45th days (D45) to detect the expression changes of miRNA-190a-5p and PHLPP1 mRNA by qRT-PCR analysis. This data showed that with the increase of VCD treating days, the expression of miRNA-190a-5p was significantly up-regulated (*p* < 0.001, Figure 4C), while the expression trend of PHLPP1 was significantly down-regulated (*p* < 0.001,

4D). Taken together, all results verified that PHLPP1 was a direct downstream target of miRNA-190-5p in POF.

### 3.3 MiRNA-190a-5p targeting PHLPP1 to promote premature ovarian failure

**FIGURE 5:**
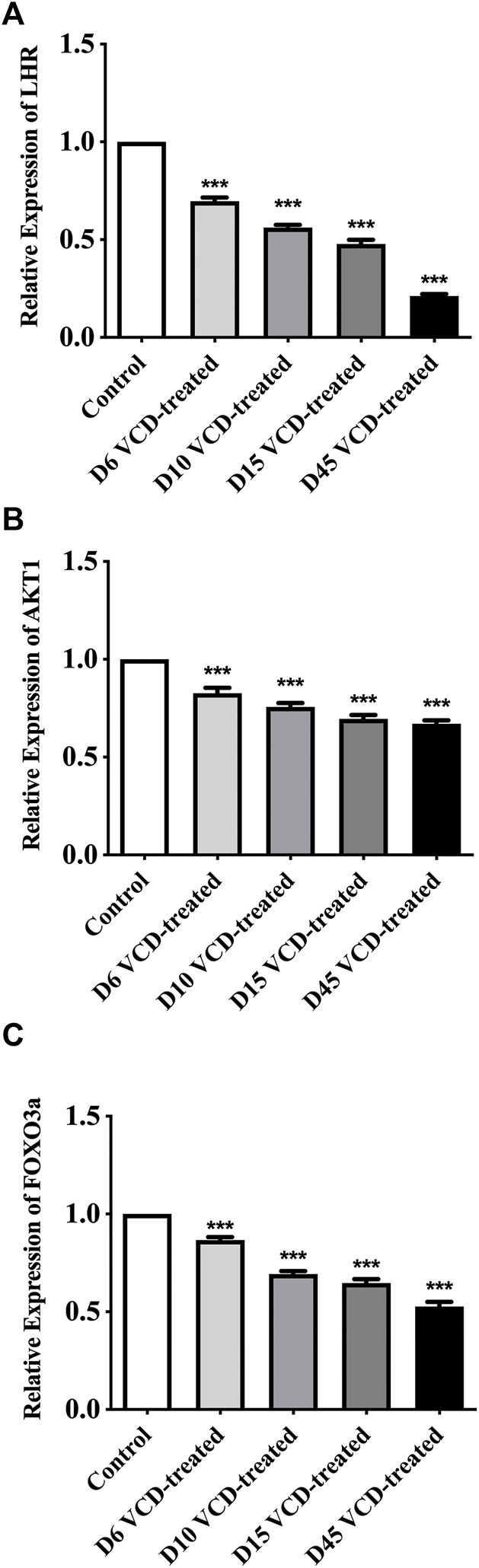
MiRNA-190a-5p targets PHLPP1 to regulate the mRNA expression levels of key genes on the primordial follicle hyperactivation signaling pathway. **(A)** LHR, **(B)** AKT1, and **(C)** FOXO3a mRNA expression during the VCD treating process (D6, D10, D15 and D45) in rat ovaries tissues were determined by qRT-PCR analysis. Data are summarized and presented as mean ± SEM from three independent experiments. ^***^ *p*＜0.001 vs. control.

**FIGURE 6:**
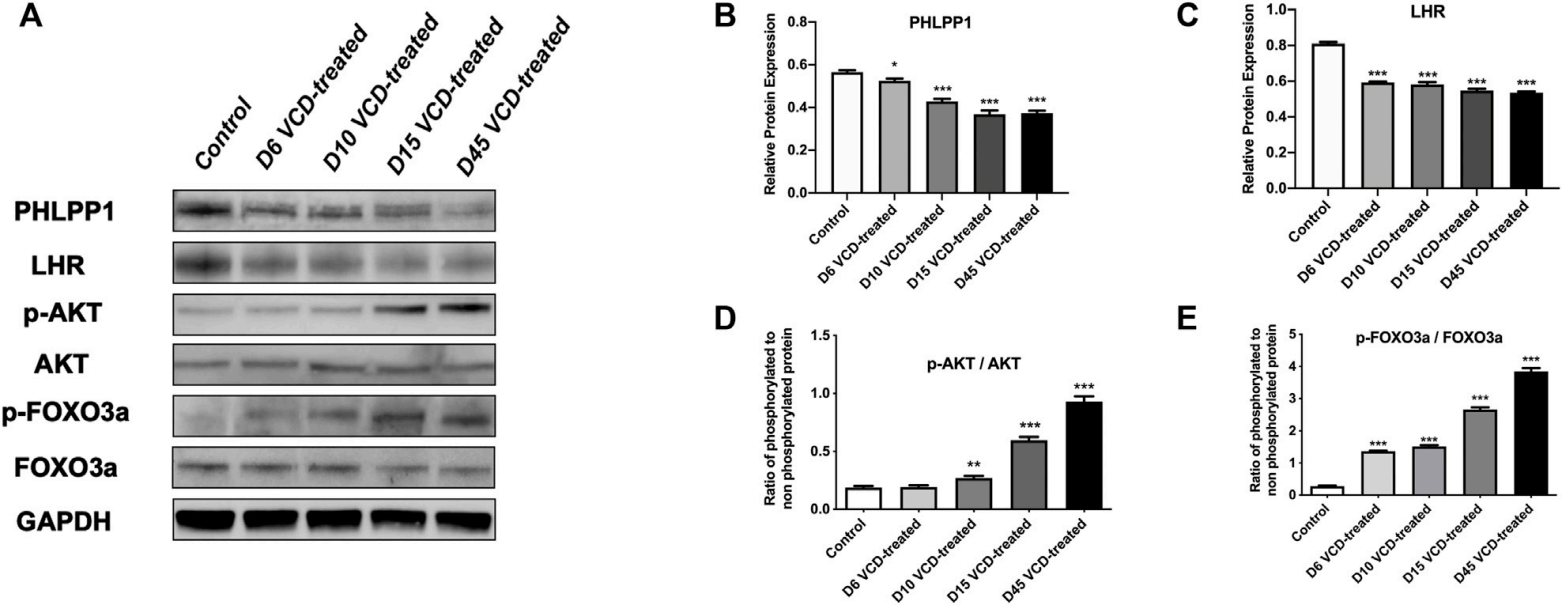
MiRNA-190a-5p targets PHLPP1 to regulate the protein expression levels of key genes on the primordial follicle hyperactivation signaling pathway. **(A)** Western blot analysis of PHLPP1, LHR, AKT, p-AKT, FOXO3a, and p-FOXO3a during the VCD treating process (D6, D10, D15, and D45) in rat ovaries tissues. GAPDH was used as a negative control. **(B)** and **(C)** Protein analysis comparing the concentrations of PHLPP1 and LHR with GAPDH used as a loading control. **(D)** and **(E)** Protein analysis comparing the concentrations of phosphorylated and total AKT, and FOXO3a with GAPDH used as a loading control, respectively. The ratio of phosphorylated to non-phosphorylated protein was calculated for each protein. Data are summarized and presented as mean ± SEM from four independent experiments. ^*^ *p*＜0.05, **p＜0.01 and ***p＜0.001 vs. control.

To study the mechanism of miRNA-190a-5p to promote POF after VCD treatment. We detected the expression level of PHLPP1 mRNA and protein and its signaling pathway mRNAs and proteins, including AKT, FOXO3a, and LHR in the VCD-treated rat ovaries tissues. As shown in Figures 5, 6, with the increase of the VCD-treated process, from the VCD-treated groups on the 6th (D6), 10th (D10), 15th (D15), and 45th days (D45), the expression of PHLPP1 mRNA and protein in each model group decreased, showing a significant downward trend (*p* < 0.05, *p* < 0.001, Figure 4D and Figure 6B). And the LHR mRNA and protein expression levels decreased, showing a significant downward trend (*p* < 0.001, Figure 5A, Figure 6C). We also found the AKT (AKT1) mRNA expression was significantly decreased (*p* < 0.001, Figure 5B) and the ratio between phosphorylated and non-phosphorylated AKT protein (p-AKT/AKT) was increased, showing a significant upward trend (*p* < 0.01, *p* < 0.001), but the difference in the D6 VCD-treated group was not significant (Figure 6D). Meanwhile, FOXO3a mRNA expression was significantly decreased (*p* < 0.001, Figure 5C), and the ratio between phosphorylated and non-phosphorylated FOXO3a protein (p-FOXO3a/FOXO3a) was increased, showing a significant upward trend (*p* < 0.001, Figure 6E).

## 4 Discussion

Premature ovarian failure (POF) is characterized by amenorrhea, infertility, low estrogen levels, excess gonadotropins, and a lack of mature follicles before age 40 (Okeke et al., 2013). The occurrence of POF is mainly related to the continuous reduction of follicles in the ovary and the abnormal development of follicles. The size of the primordial follicles and the maintenance of the resting state determine the speed of ovarian aging. Once the primordial follicles are exhausted, the ovaries will be rapidly depleted after losing their function, and the body will enter a state of menopause. Studies have shown that 4-vinylcyclohexene diepoxie (VCD), a common ovarian toxic chemical, accelerates ovarian failure by specifically activating primordial follicles, and persists after drug withdrawal characteristics of toxicity. In animal experiments, the different stages of ovarian function from weakened to failure can be simulated by controlling the dosage and modeling time, so it can be widely used in the study of decreased ovarian reserve, POF, and menopause-related diseases (Hoyer et al., 2001; Frye et al., 2012; Kappeler and Hoyer, 2012). In addition, compared with other modeling methods, VCD only affects gonadal tissues such as ovaries, and hardly affects other tissues or organs (Tamura et al., 2009). And some experiments have confirmed that VCD can induce POF in experimental animal models (Hoyer et al., 2001; Wright et al., 2011). Therefore, in the early stage of our study (Kuang et al., 2014; Li et al., 2014), VCD was used to successfully induce the POF animal model.

Based on our previous study, we still used the same approach to establish the POF rat model in the present study. Even for the purpose of collecting tissues during VCD modeling (i.e., during primordial follicular hyperactivation). We tested blood samples and collected rat ovarian tissue after sacrificing rats at consecutive time points of VCD injection. Hormone level is an important and sensitive indicator of ovary function. The E_2_, FSH, and LH levels are classical criteria for POF (Steiner, A. Z., 2013; Nie, X et al., 2018). Clinical studies showed that patients in the POF group were found to have significantly higher FSH and LH serum concentrations and lower E_2_ serum levels when compared to healthy controls (Podfigurna, A et al., 2018; Qi et al., 2022). Our hormone level results are consistent with our previous findings (Kuang et al., 2014; Li et al., 2014). However, it is worth noting that in our result, we found that LH was the hormone with early differential changes in the VCD-treated process, that is, from the 6th day of treatment. In addition, some studies have shown that the change in LH level is an important link to trigger follicle ovulation in the process of follicle development (Holesh et al., 2021). Therefore, we speculate that the abnormal change in LH level caused by VCD modeling will damage the development of follicles and ovulation function.

In this study, we additionally observed the changes in serum Anti-Mullerian hormone (AMH) levels in rats during VCD-treated days. And we found that the AMH level decreased with the increase of VCD-treated time. Previous studies have shown that chemicals can destroy growing follicles, which indirectly causes a reduction in the primordial follicular pool (Wang et al., 2019), as the destruction of growing follicles leads to a decrease in AMH, which activates the primordial follicles, and an increase in follicular granulosa cells to produce AMH to inhibit further primordial follicular activation (Durlinger et al., 1999). Thus, the activation and recruitment of primordial follicles may be accelerated in mice molded using the chemical induction method due to the sudden loss of growing follicles (Baker et al., 2011). Another study showed that when ovarian function is impaired and the number of growing follicles decreases leading to a decrease in AMH, its function of maintaining the dynamic balance between follicular atresia and follicular recruitment decreases, which induces the primordial follicles to be overactivated, and this dynamic balance, if not restored, can eventually lead to POF (Moolhuijsen and Visser, 2020). Therefore, our results speculate that the overactivation of primordial follicles becomes more pronounced as the days of VCD administration increase. Hence, it is therefore important to seek novel biomarkers of primordial follicle hyperactivation.

Based on successfully replicating the POF animal model by VCD-treated, our previous study detected the most significantly differentially expressed 6 miRNAs between POF and normal rats’ ovaries tissues by using the rat genome-wide miRNA expression profile chip technology, including miRNA-190a-5p, miRNA-98-5p, miRNA-29a-3p, miRNA-144-5p, miRNA-27b-3p and miRNA-151-5p (Kuang et al., 2014). Other previous studies have shown that miRNAs are involved in the maturation of mouse oocytes and the development of follicles (Dehghan et al., 2021). Hence, our study continuously monitored the expression changes of these 6 miRNAs from the first day of VCD-treated and screened out potential biomarkers that can be used in the early stage of POF disease. In our study, we found that miRNA-190a-5p was abnormally upregulated at the early stage of VCD injection, i.e., the 6th day (D6), and showed a significant upward trend with the increase of VCD-treated days. Therefore, we speculated that miRNA-190a-5p might serve as a biomarker in the early stage of POF. Usually, we also know that miRNAs exert their functions by degrading mRNAs expression (Othman and Nagoor, 2019; Tian et al., 2019). Hence, we used the publicly available databases to seek the potential targets of miRNA-190a-5p. Previous research reported that miRNA-190a directly inhibits the PH domain leucine-rich repeat protein phosphatase (PHLPP) (Yu et al., 2014). Likewise, in this study, we firstly predicted by database and then verified that miRNA-190a-5p negatively regulates its downstream target gene PHLPP1 at the early stage of POF, i.e., from the 6th day of VCD-treated POF, by double-luciferase reporter gene assay and qRT-PCR assay in rat ovarian tissues. It is thus hypothesized that miRNA-190a-5p may play a role in promoting premature ovarian failure by targeting PHLPP1.

PHLPP1 is a tumor suppressor protein that inactivates the kinase AKT through Ser437 dephosphorylation (Gao et al., 2005). A recent study showed that trivalent arsenic (A^3+^) induces the expression of miR-190 in human bronchial epithelial cells, which binds the 3′UTR of the PHLPP transcript, decreasing PHLPP protein levels. Subsequently, AKT activation and phosphorylation levels were increased (Beezhold et al., 2011). In addition, in chemical environment induced POF models, several studies have confirmed the presence of enhanced AKT kinase signaling pathways and lead to primordial follicular hyperactivation (Sobinoff et al., 2011; Sobinoff et al., 2012). It has even been shown that after direct or indirect activation of AKT, phosphorylated AKT can activate phosphorylation of a series of downstream proteins (FOXO3a, BCL-2, etc.), which can promote follicular cell growth and proliferation (Makker et al., 2014). Moreover, AKT activation by upstream molecules has been reported to hyperphosphorylated FOXO3a, transport it out of the nucleus, and stimulate primordial follicle initiation (John et al., 2008). Therefore, FOXO3a can also be used as a marker to determine if the primordial follicle is activated (Yan et al., 2018). Thus, in this study, we used qRT-PCR assay and Western Blot analysis to detect the effects of VCD modeling on the expression of PHLPP1, AKT, and FOXO3a mRNA and the phosphorylation levels of AKT and FOXO3a proteins in rat ovarian tissues, and observed that as the time of VCD modeling increased, the expression levels of PHLPP1 mRNA and protein decreased along with the expression levels of AKT and FOXO3a mRNA and proteins, while the phosphorylation levels of AKT and FOXO3a proteins showed a significant increase. Therefore, we hypothesized that the abnormal expression of miRNA-190a-5p was upregulated in VCD-treated rats followed by downregulation of PHLPP1, which further activated AKT and FOXO3a proteins on AKT-FOXO3a signaling pathway, thus hyperactivation primordial follicles leading to the development of POF.

As mentioned previously in this study the latest finding was that LH was the earlier hormone to show differential changes during the VCD-treated POF rat model and was significantly differentially expressed almost the same day as miRNA-190a-5p. It has been reported that LH itself induces the expression of luteinizing hormone receptor (LHR) and that the pulsatile release of LH maintains the levels of LHR and steroid hormone synthase (Plakkot et al., 2018). However, the experimental elevation of LH levels can desensitize hormonal signaling and lead to the downregulation of LHR expression (Dufau, 1998). It has been shown that mutations in the LHR gene lead to abnormal LHR function and failure to properly receive LH and stimulate hormonal signaling to the second messenger, resulting in impaired follicular maturation in the ovary, anovulation, delayed puberty, amenorrhea, infertility, with typical POF symptoms (Dixit et al., 2010). In addition, it has been shown that AKT knockout in mice is less responsive to LH processing, leading to a reduction in the number of primordial follicles in mouse ovaries (Maidarti et al., 2020). A recent study showed that cisplatin-induced primordial follicular hyperactivation in mice led to a significant reduction in the number of LHR expressions during POF (Chang et al., 2015). In our research, we found that the gene and protein expression of LHR in rat ovarian tissues showed a decreasing trend with the increase of VCD modeling time by qRT-PCR assay and Western Blot analysis. Therefore, we speculate that one of the alternative mechanisms by which VCD-induced miRNA-190a-5 promotes POF in rats may be that VCD induces miRNA-190a-5p to downregulate PHLPP1 after upregulation of abnormal expression at an early stage, which subsequently activates AKT, causing a decrease in AKT expression, leading directly or indirectly to an abnormal elevation of LH hormone and decrease in LHR receptor expression level, thus hyperactivation primordial follicles and ultimately leading to POF.

## 5 Conclusion

In this study, we found that miRNA-190a-5p may become a potential biomarker for early screening of POF, and it can continuously hyperactivation primordial follicles in rats by targeting the expression of PHLPP1 and key proteins in the AKT-FOXO3a and AKT-LH/LHR pathways.

## Data Availability

The original contributions presented in the study are included in the article/Supplementary Material, further inquiries can be directed to the corresponding authors.
